# Supplementary material for: Disruption of the autoinhibited state primes the E3 ligase parkin for activation and catalysis
Source: EMBO J. 2015 Aug 7;34(20):2506–21. doi: 10.15252/embj.201592337 (PMC4609183; doi:10.15252/embj.201592337)
Supplement: Supplementary file 1 — Appendix [file embj0034-2506-sd1.pdf]

Disruption of the autoinhibited state primes the E3 ligase parkin for activation and catalysis

Atul Kumar[1+], Jacob D. Aguirre[2+], Tara E.C. Condos[2+], R. Julio Martinez-Torres[1+], Viduth K. Chaugule[1], Rachel Toth[1], Ramasubramanian Sundaramoorthy[3], Pascal Mercier[2], Axel Knebel[1], Donald E. Spratt[2], Kathryn R. Barber[2], Gary S. Shaw[2*], Helen Walden[1*]

Appendix
Appendix figures S1-S7

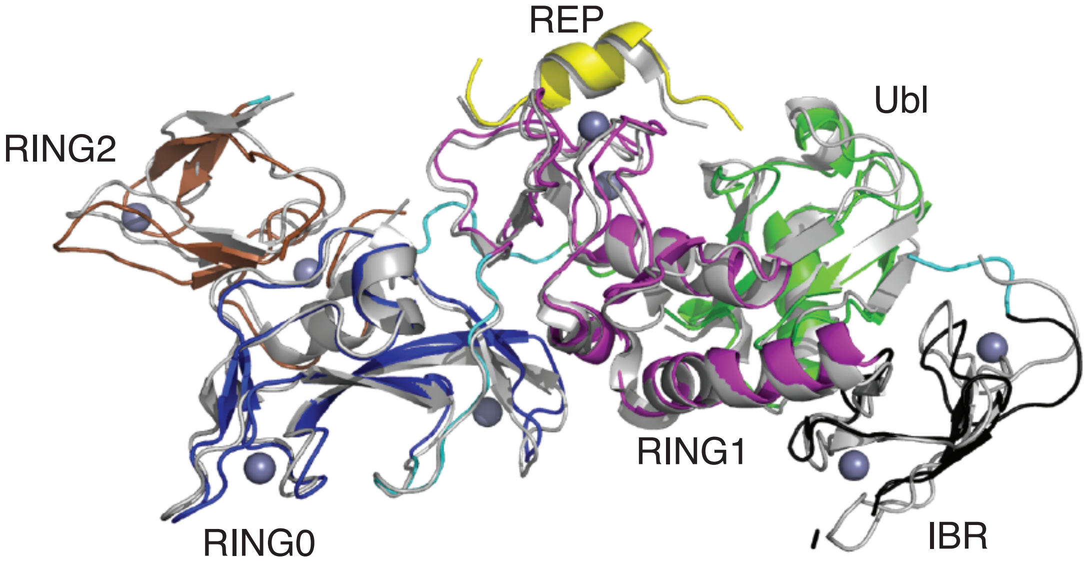

Appendix Figure S1. Comparison of the global folds for Parkin. Superposition of UblR0RBR Parkin (coloured) with full-length rat Parkin (grey), pdb code 4K95 (Trempe et al., 2013). Each domain is labelled, zinc atoms are shown in spheres. The orientation is equivalent to Figure 1C top panel.

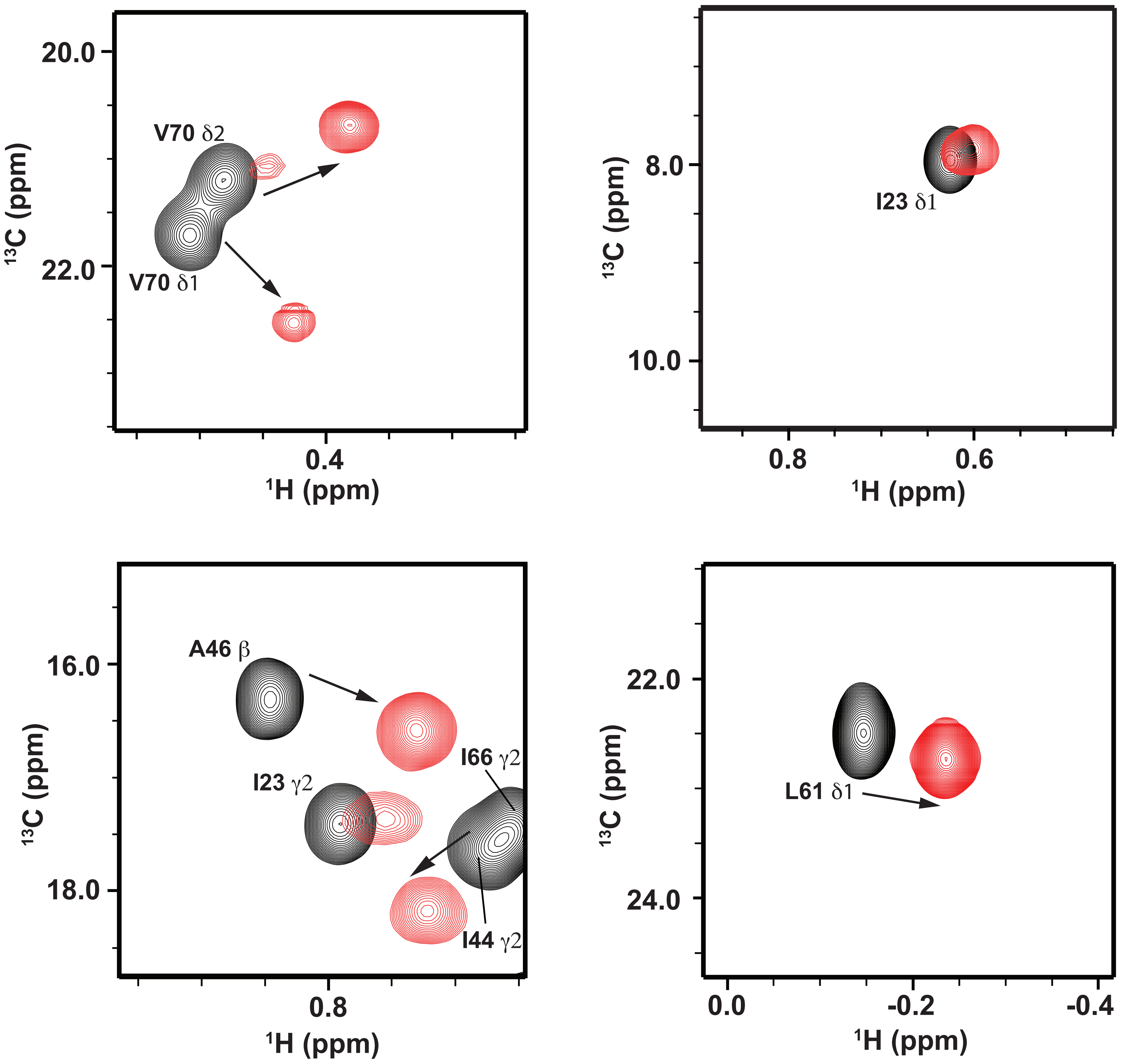

Appendix Figure S2. Ubl interactions with R0RBR Selected methyl regions for 600 MHz ^{1}H-^{13}C HMQC spectra of 150 μM ^{13}C-labelled Ubl domain alone (black contours) and in the presence of 1.5 molar equivalents ^{2}H,^{12}C,^{15}N-labelled R0RBR (red contours). Based on the crystal structure, chemical shift perturbations for A46, L61 and I66 face the tether region following the IBR domain where A383-A390 were not observed indicating these residues in the Ubld domain transiently interact with this region.

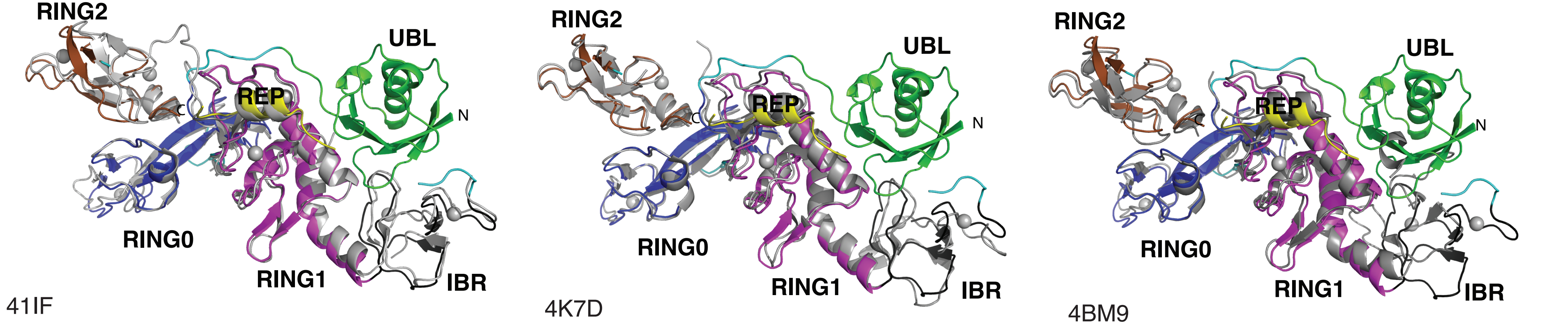

Appendix Figure S3. The global conformation of parkin is similar in the presence or absence of the Ubl domain. UblR0RBR parkin is superimposed on human high-resolution crystal structures of human R0RBR parkin (left), rat R0RBR parkin (middle), and human R0RBR parkin (right) generated by different labs (Riley et al., 2013; Trempe et al., 2013; Wauer and Komander, 2013; respectively). Domains are labelled and in each image the UblR0RBR structure is coloured according to domain while the R0RBR structures are grey. The orientation is as Fig 1C, bottom panel.

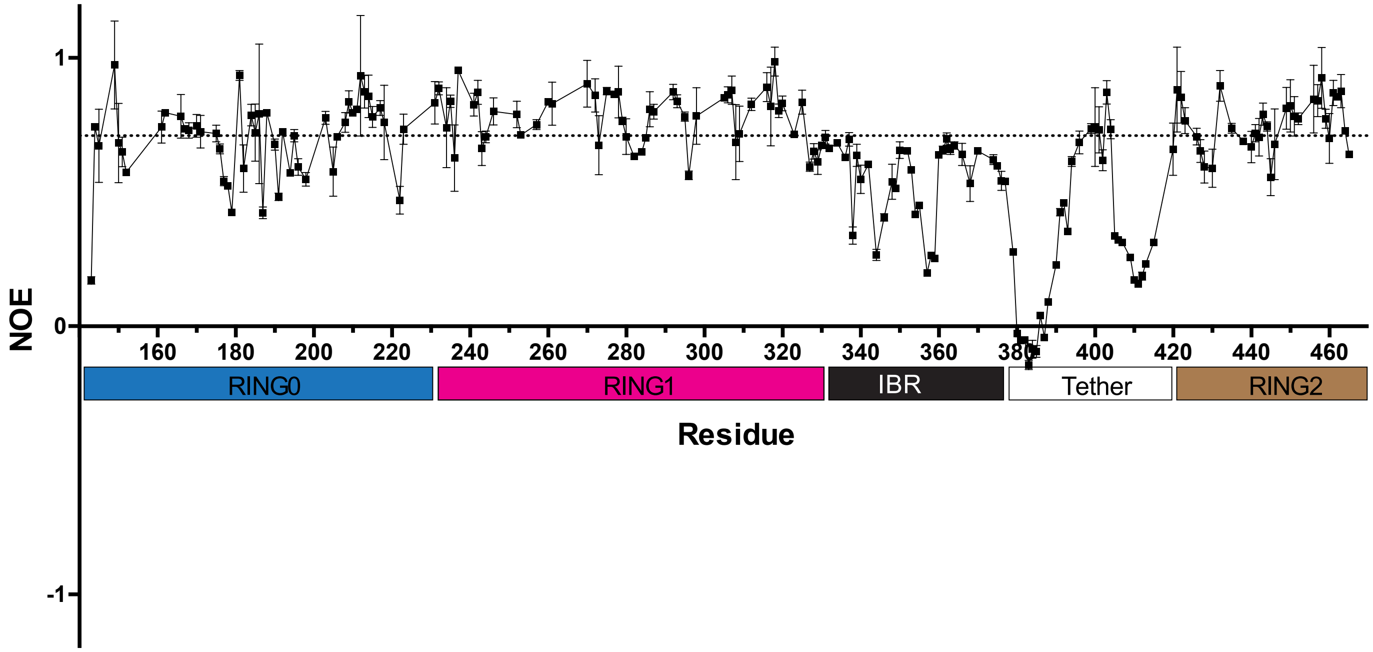

Appendix Figure S4. ^{1}H-^{15}N Heteronuclear NOE values of R0RBR parkin. Only resolved resonances in the ^{1}H-^{15}N HSQC spectrum were selected for analysis. The values plotted are the average of two independent experiments (indicated by error bars) at 600MHz. The average NOE of the data within 1SD of the mean was 0.71, indicated by a dashed line. Decreased NOE values in the IBR and tether domains are indicative of increased mobility in the protein backbone.

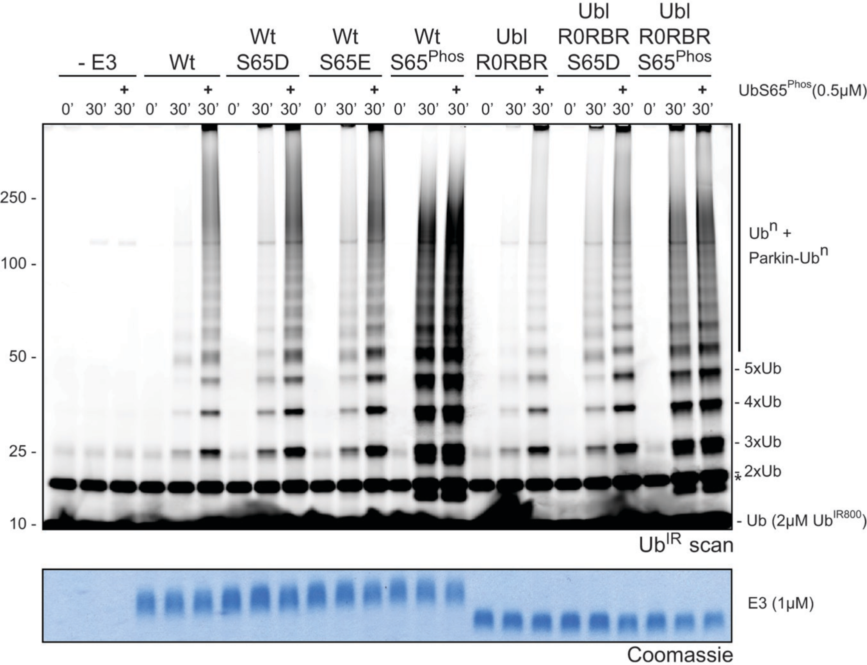

Appendix Figure S5. Comparison of ubiquitination activity of parkin phosphomimetics, phosphoparkin, and the crystallised constructs.

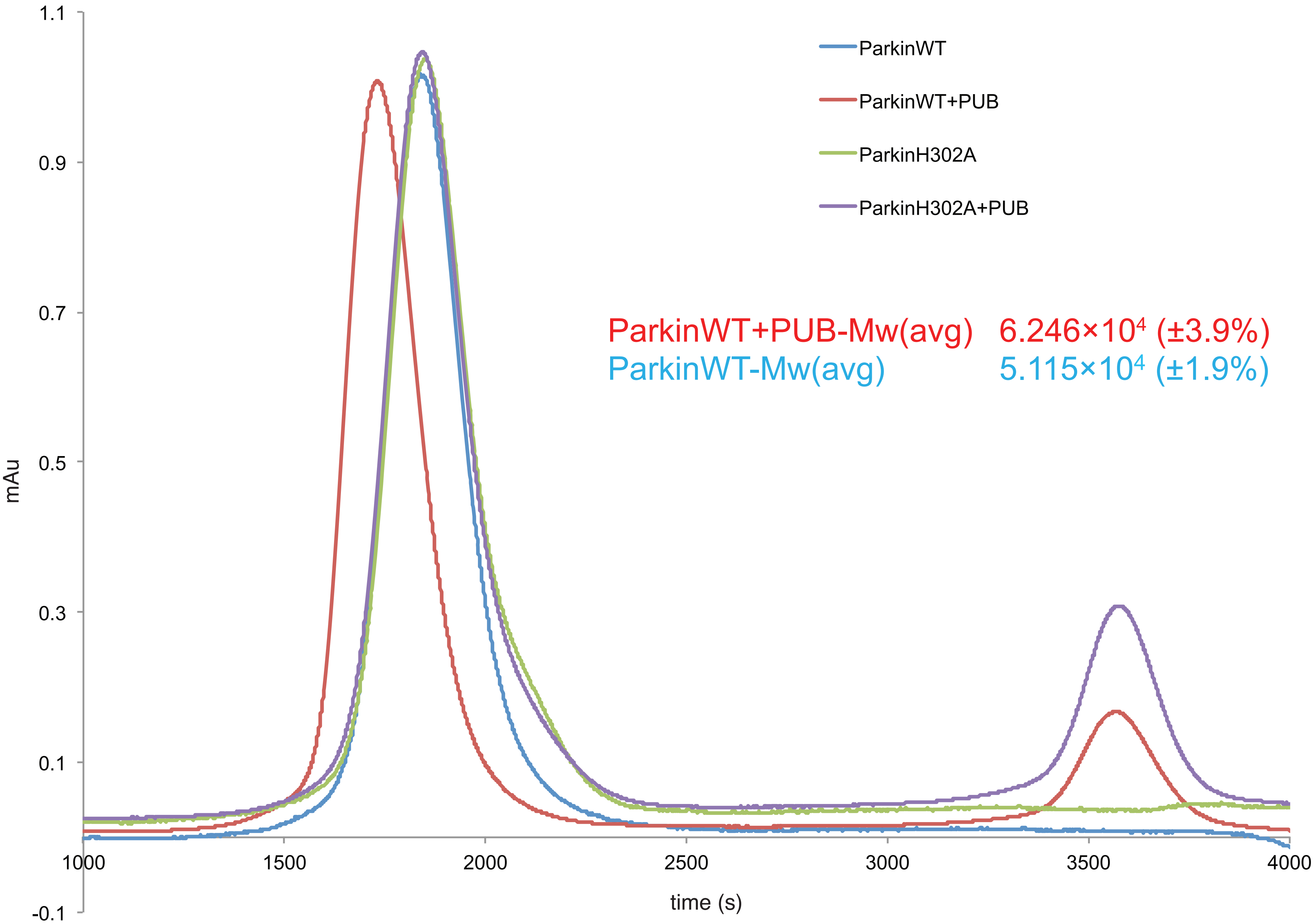

Appendix Figure S6. Size-exclusion chromatography followed by Multi-angle light scattering analysis of Parkin interaction with phosphoubiquitin. Wild-type Parkin (blue) forms a complex with phosphoubiquitin (red). In contrast H302A-Parkin (green) does not complex with phosphoubiquitin (purple).

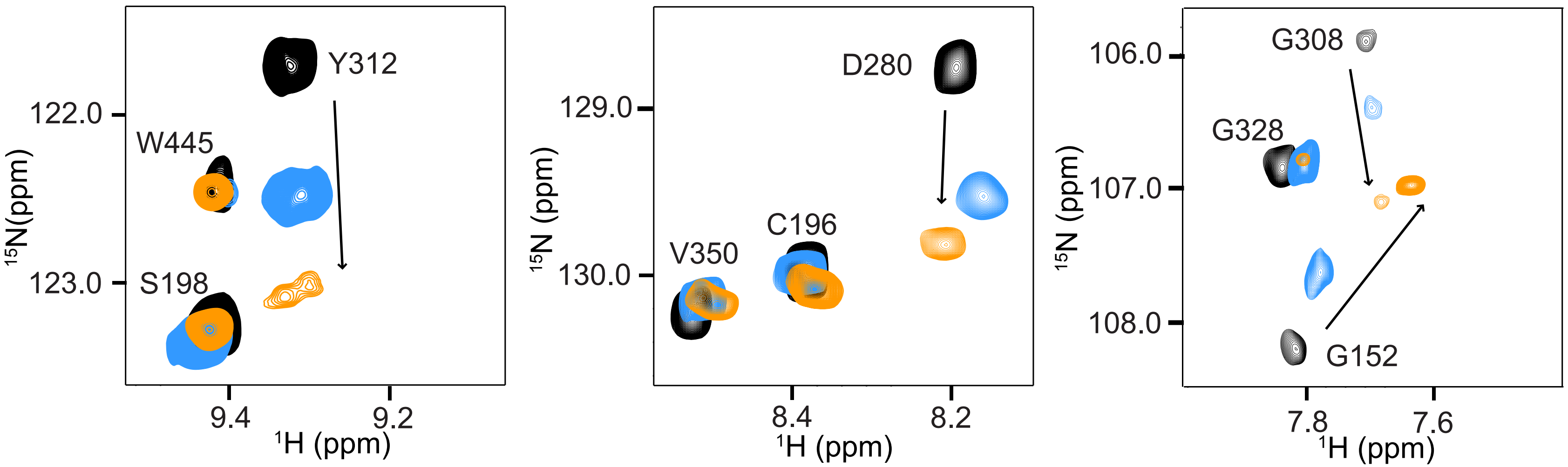

Appendix Figure S7. Selected regions of ^{1}H-^{15}N TROSY spectra. The data shows chemical shift changes for R0RBR (black contours) upon addition of either S65EUb (blue contours) or pUb (orange contours). The spectra highlight changes that occur for Y312 (RING1 helix H3), D280 (RING1 β16-β17), and G308 and G152 (either side of RING0/RING1 hinge).
