# Supplementary figures and images for: Disruption of the autoinhibited state primes the E3 ligase parkin for activation and catalysis
Source: EMBO J. 2015 Aug 7;34(20):2506–21. doi: 10.15252/embj.201592337 (PMC4609183; doi:10.15252/embj.201592337)

## Expanded View Figures

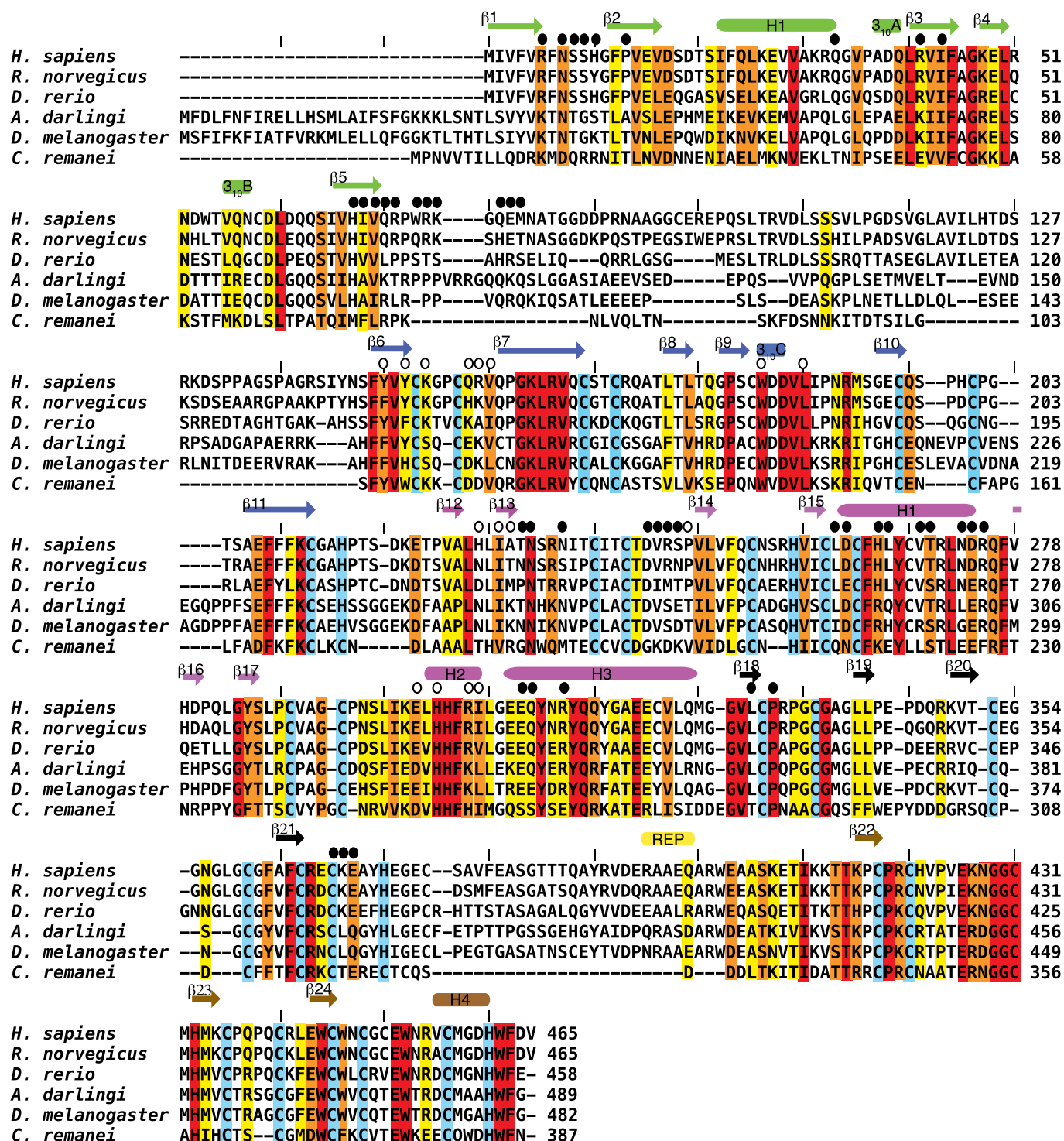

Supplement: Supplementary file 2 — Expanded View Figures PDF [file embj0034-2506-sd2.pdf]
